# A Supplementary Comment on “Reliability and Validity of a Questionnaire for Assessment of Physical Activity in Epidemiological Studies” Published in Journal of Epidemiology, 1998

**DOI:** 10.2188/jea.12.54

**Published:** 2007-11-30

**Authors:** Hiroyuki Shimizu

In recent years, much attention has been paid to the relationships between physical activity and chronic diseases. Physical activity questionnaire has been often used to assess these associations in epidemiological studies. However, the validity of the questionnaire has been seldom tested in a Japanese population. We conducted a validation study of a self-administered physical activity questionnaire (PAQ), which was designed to assess both total energy expenditure and energy expenditure by physical activity at work and at leisure in a large scale cohort, and reported the results in the Journal of Epidemiology, 1998; 8:152-159.

In the article, to evaluate the validity of PAQ we calculated the correlation coefficient between the total energy expenditure as well as energy expenditure by physical activity estimated from record in PAQ and that in Calorie Counter (CC). CC is a small machine designed to detect the speed and the acceleration rate along the vertical axis at the waist during body movement. Estimated daily energy expenditures from PAQ were 2,171 kcal/day for men and 1,729 kcal/day for women, and those from CC were 2,274 kcal/day and 1,782 kcal/day for men and women, respectively. Correlation coefficients between the two methods for daily energy expenditures were 0.57 for 49 men and 0.68 for 32 women. Furthermore, correlation coefficients for energy expenditure by physical activity per week were 0.69 and 0.69 for men and women, respectively. Based on these results, we summarized that our PAQ has adequate levels of validity in the assessments of daily energy expenditure and weekly physical activity.

However, our discussion was inadequate to point out the problems. We did not well consider the effect of basal metabolism (BM) for the analysis of daily energy expenditure although BM is the major components of the expenditure and BM is mostly depended on area of body surface or height and weight.

The correlation coefficients between daily energy expenditures obtained from PAQ and CC were 0.5 or over in both sexes. However, body weight was highly correlated with both PAQ (r = 0.83 for men and r = 0.81 for women) and CC (r = 0.63 for men and r = 0.78 for women). Thus, one can estimate daily energy expenditure better using weight alone than using the questionnaire.

On the other hand, the correlation coefficients between energy expenditure by physical activity obtained from PAQ and body weight were 0.17 for men and 0.56 for women. To minimize the effect of body weight on the correlation between the two methods, we recalculated the correlation coefficients using variables of PAQ and CC after divided by body weight. The correlation was still high for both sexes (r = 0.69; p = 0.0001 for men and r = 0.62; p = 0.0002 for women).

To evaluate the reliability or reproducibility of the questionnaire, we conducted a test-retest study by comparing the total energy expenditure and energy expenditure by physical activity estimated from the repeated PAQs with one-year interval among 95 men and 119 women. However, the energy expenditure depending on body weight was not appropriate as a variable for the study. We estimated the metabolic equivalents (METs) from the repeated PAQs and obtained the high correlation coefficients between the two METs with one-year interval (r = 0.51 for men, p = 0.0001 and r = 0.44 for women, p = 0.0001).

In summary, we conclude that our physical activity questionnaire has adequate level of validity and reliability to measure weekly physical activity, but not to measure daily energy expenditure, in epidemiological studies.

(On behalf of the authors)

